# Risk factors and clinical significance of neurodegenerative co-pathologies in symptomatic cerebral small vessel disease

**DOI:** 10.1007/s00415-025-13087-z

**Published:** 2025-04-18

**Authors:** Philipp Arndt, Malte Pfister, Valentina Perosa, Hendrik Mattern, Jose Bernal, Anna-Charlotte John, Marc Dörner, Patrick Müller, Rüdiger C. Braun-Dullaeus, Cornelia Garz, Christopher Nelke, Alma Kokott, Robin Jansen, Michael Gliem, Sven G. Meuth, Solveig Henneicke, Stefan Vielhaber, Katja Neumann, Stefanie Schreiber

**Affiliations:** 1 Department of Neurology, Otto-Von-Guericke University, Leipziger Str. 44, 39120 Magdeburg, Germany; 2 German Center for Neurodegenerative Diseases (DZNE) Within the Helmholtz Association, Magdeburg, Germany; 3 J. Philip Kistler Stroke Research Center, Massachusetts General Hospital/Harvard Medical School, Boston, MA USA; 4 Biomedical Magnetic Resonance, Faculty of Natural Sciences, Otto-Von-Guericke University, Magdeburg, Germany; 5 Center for Clinical Brain Sciences, The University of Edinburgh, Edinburgh, UK; 6 Department of Consultation-Liaison-Psychiatry and Psychosomatic Medicine, University Hospital Zurich, University of Zurich, Zurich, Switzerland; 7 Department of Cardiology, Otto-Von-Guericke University, Magdeburg, Germany; 8 German Center for Mental Health (DZPG), Magdeburg, Germany; 9 Department of Neurology, Heinrich-Heine-University, Düsseldorf, Germany

**Keywords:** Cerebral small vessel disease, Deep perforator arteriopathy, Cerebral amyloid angiopathy, ATN classification, Alzheimer's disease pathology, Non-AD pathological change

## Abstract

**Background:**

Cerebral small vessel disease (CSVD) often coexists with neurodegenerative pathologies, yet their role remains underexplored. This study aims to determine their prevalence, risk factors, and cognitive effects in patients with deep perforator arteriopathy (DPA) or cerebral amyloid angiopathy (CAA) using the biomarker-based ATN classification.

**Methods:**

In this cross-sectional study 186 patients (median age 75 years, 41% females, 111 with probable CAA, 75 with DPA) underwent MRI for analysis of CSVD severity and etiology, and lumbar puncture for analysis of cerebrospinal fluid amyloid-β 42/40 ratio, phosphorylated-tau, total-tau and neurofilament light. ATN profiles were related to clinical characteristics, MRI markers and cognitive performance in multivariate regression models.

**Results:**

Among CSVD patients, 30% had normal biomarkers (A-T-N-), 33% were within the AD pathology continuum (A + T ± N ± : 47% in CAA vs. 13% in DPA, p < .001), and 37% showed non-AD pathological changes (A-T ± N + : 53% in DPA vs. 25% in CAA, p < .001). The AD pathology continuum was associated with a severe lobar hemorrhagic phenotype and cognitive impairment, while non-AD pathological change was related to CSVD severity, history of stroke and similarly cognitive impairment. Both pathological ATN profiles were further related to lower MMSE scores (A + T ± N ± : B = − 3.3, p = .006; A-T ± N + : B = − 2.7, p = .021).

**Conclusions:**

Using biomarkers, this study confirms in vivo that CSVD frequently co-occurs with neurodegenerative pathologies, exerting detrimental effects on cognitive health.

**Supplementary Information:**

The online version contains supplementary material available at 10.1007/s00415-025-13087-z.

## Background

*Postmortem* neuropathologic studies revealed that the co-occurrence of cerebral small vessel disease (CSVD) and Alzheimer’s disease (AD) or further neurodegenerative pathology is common and accelerates cognitive impairment, compared to isolated pathologies [1–4].

In clinical practice, the characterization of concurrent brain pathologies in vivo remains rare, as typically, primary symptomatic pathologies receive focus in care. CSVD diagnosis relies on magnetic resonance imaging (MRI) detection of non-hemorrhagic and hemorrhagic lesions, with heterogeneous clinical presentation and a lack of disease-specific biofluid markers [5] Conversely, AD dementia diagnosis is based on disease-specific biofluid markers for amyloid-beta (Aβ) and phosphorylated-tau (pTau), alongside a specific cognitive profile [6]. Integrating the ATN classification, a biomarker-based scheme for AD pathology diagnosis into the MRI-based CSVD framework, facilitates the identification of individuals with isolated or mixed brain pathologies.

In vivo studies have confirmed the significance of concurrent CSVD in AD dementia patients. Compared to cognitively unimpaired controls, they demonstrated more frequently CSVD lesions on MRI, which accelerated the trajectory of cognitive decline [7–12].

In contrast, less is known about mixed pathologies in patients with symptomatic CSVD. Some studies investigated concurrent Aβ or tau pathology using positron electron tomography (PET) imaging [13–16], but these studies comprise small sample sizes or lack precise clinical and neuroradiological characterization of the underlying CSVD etiology. Further, the entire ATN classification has not been fully applied in symptomatic CSVD to investigate AD (A + T ± N ±) and non-AD neurodegenerative (A–T ± N +) co-pathologies.

We aimed to overcome these knowledge gaps integrating MRI and fluid biomarkers in a clinical cohort of 186 patients, including patients with symptomatic deep perforator arteriopathy (DPA) and cerebral amyloid angiopathy (CAA), to investigate prevalence of AD and non-AD neurodegenerative co-pathologies, their risk factors and relevance for global cognition in vivo.

## Methods

### Study population and inclusion criteria

This retrospective study included symptomatic patients from a prospectively curated CSVD database, which registers symptomatic individuals with MRI-confirmed cerebral microbleeds (CMB) and associated clinical data at the Department of Neurology, Otto-von-Guericke University Magdeburg, covering the period from October 2010 to February 2024. Out of 711 patients in the CSVD database who had MRI data available, 186 also had CSF biomarker data and were included in this study (75 DPA, 111 CAA). Only those with a complete CSVD assessment according to STRIVE were included.

Consequently, the following inclusion criteria were applied: (i) patients had to have both MRI and CSF biomarker data available, with all necessary biomarkers and imaging sequences for diagnostic assessment, (ii) CSVD patients required MRI-confirmed DPA, with the presence of deep CMB, or probable CAA, according to the Boston criteria 2.0 [17]. Patients were excluded if MRI quality was insufficient for a complete CSVD assessment based on STRIVE criteria, and if CSF biomarker data were incomplete so that the ATN status could not be determined.

### Clinical data and neuropsychological assessment

Patients were characterized with regard to clinical phenotype at presentation (cognitive impairment, history of stroke, gait disturbances, seizure or a mixture of them), demographics (age, sex, years of education (available for n = 124, 67%)) and vascular risk factors (available for n = 175, 94%). Global cognition was determined through Mini Mental State Examination (MMSE, available for n = 92, 49%) (for details see Supplementary material).

### Cerebrospinal fluid

Lumbar puncture was performed as part of the diagnostic workup for cognitive impairment, gait disturbances, stroke symptoms, seizures, or a combination of these symptoms. CSF samples were centrifuged at 4 °C, aliquoted, and stored at − 80 °C until analysis. Biomarker levels were determined using ELISA kits (until 12/2019: Innotest Aβ40, Innotest Aβ42, Innotest pTau, Innotest hTauAg, Innogenetics, Ghent, Belgium; NfL Umandiagnostics, Sweden) or automated immunoassays (LUMIPULSE® G600 II, Fujirebio Inc., Japan, from 01/2020). Locally established thresholds were as follows: 0.50 for Aβ_42/40_ ratio, 70 pg/mL for phosphorylated Tau (pTau), 350 pg/mL for total Tau (tTau), and 3643 pg/mL for neurofilament light chain (NfL) using ELISA kits, and 0.69 for Aβ_42/40_ ratio, 56 pg/mL for pTau, and 404 pg/mL for tTau using immunoassays. CSF biomarker levels of AD pathology (Aβ_42/40_ ratio, pTau) and neurodegeneration (tTau and NfL) were used to categorize patients as A-T-N- (normal biomarker status), A+T± N± (AD pathology continuum), A-T±N+ (non-AD pathological change). None of the patients was classified A-T+N.

### MRI acquisition and analysis

For analysis, clinical 3T MRI (n = 90, 48%) or 1.5T MRI (n = 96, 52%) was used to quantify intracerebral hemorrhage (ICH), CMB, white matter hyperintensities (WMH) and lacunes according to the CSVD Standards for Reporting Vascular Changes on Neuroimaging (STRIVE) criteria [5] by one trained investigator (M.P.) blinded to demographic and clinical data. Global CSVD severity was determined through an ordinal MRI CSVD sum score ranging from 0 to 5 points, in which one point was allocated for the presence of lacunes, 1–4 CMB and moderate WMH (WMH grade 3–4 according to Fazekas [18]) and two points were allocated for ≥ 5 CMB and severe WMH (WMH grade 5–6), respectively (for details see Supplementary material).

### Statistical analysis

Results of continuous variables were expressed as median (IQR) or mean (SD), as appropriate; results of categorical variables were expressed as proportions. Intergroup comparisons were performed in univariate analyses, using the χ^2^-test, 2-sample t-test or Mann-Whitney U-test. Risk factors of pathological ATN profiles in CSVD patients were investigated in two steps: first, univariate tests were performed to compare clinical and MRI characteristics of patients within the AD pathology continuum (A+T±N±) or those with non-AD pathological changes (A-T±N+) to patients with normal biomarkers (A-T-N-), respectively. To explore independent predictors of these pathological ATN profiles, we performed multivariable logistic regression analyses in a stepwise, forward-elimination approach (minimal adjusted model), based on significant variables from univariate tests (p < 0.05). Age and sex were forced to be included in the models. Subsequently, the regression analysis was repeated for DPA and CAA subgroups to test the generality of the risk factors. Finally, a multivariate linear regression model was conducted to examine associations between pathological ATN profiles and MMSE score in CSVD, accounting for age, sex, number of vascular risk factors, history of stroke and MRI CSVD sum score. (Adjusted) Significance level was set at 0.05 for all analyses. IBM SPSS Statistics 24.0 software was used for all analyses.

## Results

We included 186 patients with a median age of 75 years (IQR 67–80), of whom 76 (41%) were female. Diagnostic groups included n = 75 (40%) patients with DPA and n = 111 (60%) patients with probable CAA. Patients presented most frequently with cognitive impairment (n = 101, 54%), history of stroke (n = 83, 45%), gait disturbances (n = 55, 30%) and seizure (n = 42, 23%). Key characteristics of the cohort and statistical group comparisons are shown in Table 1.

**Table 1:** Inter-group comparison of patient characteristics

	DPA n = 75	CAA n = 111	Statistical analysis
*Demographics*			
Age [years]	70 (60–78)	76 (72–81)	**Z = − 4.4, p < .001**
Female sex	31 (41%)	45 (41%)	χ2 = 0.0, p = .914
Education [years]^a^	13 (11–16)	13 (11–16)	Z = 0.3, p = .760
MMSE^b^	24.6 (3.9)	22.6 (4.8)	T = **− **2.1, **p = .043**
*Vascular risk factors*			
Hypertension	68/72 (94%)	90/103 (87%)	χ2 = 2.4, p = .120
Dyslipidemia	35/72 (49%)	41/103 (40%)	χ2 = 1.3, p = .248
Type 2 diabetes	18/72 (25%)	33/103 (32%)	χ2 = 1.0, p = .313
*CSVD pathology on MRI*			
CSVD MRI sum score	4 (3–5)	3 (3–4)	**Z = 3.5, p < .001**
Lobar ICH on MRI	11 (15%)	27 (24%)	χ2 = 2.6, p = .109
Deep ICH on MRI	4 (5%)	0 (0%)	**χ2 = 6.1, p = .049**
Presence of cSS	10 (13%)	23 (21%)	χ2 = 1.7, p = .196
Number of lobar CMB	7 (3–20)	5 (2–18)	Z = 0.5, p = .648
Log (number of lobar CMB)	0.90 (0.60–1.32)	0.78 (0.48–1.28)	Z = 0.5, p = .648
Number of deep CMB	3 (1–6)	0 (0–0)	**Z = 13.0, p < .001**
Presence of lobar lacune	33 (44%)	33 (30%)	χ2 = 3.8, p = .051
Presence of deep lacune	31 (41%)	24 (21%)	**χ2 = 9.0, p = .003**
Periventricular WMH	3 (2–3)	3 (2–3)	Z = 1.4, p = .170
Deep WMH	3 (2–3)	2 (2–3)	Z = 1.8, p = .070

Data are represented as median (IQR), mean (SD) or n (%). Significant p-values are marked in bold

^a^ available for n = 124 (67%), ^b^ available for n = 92 (49%)

*CAA* cerebral amyloid angiopathy, *CMB* cerebral microbleeds, *cSS* cortical superficial siderosis, *CSVD* cerebral small vessel disease, *DPA* deep perforator arteriopathy, *ICH* intracerebral hemorrhage, *MMSE* Mini Mental State Examination, *MRI* magnetic resonance imaging, *WMH* white matter hyperintensities

### One third of CSVD patients is within the AD pathology continuum

**Fig. 1:**
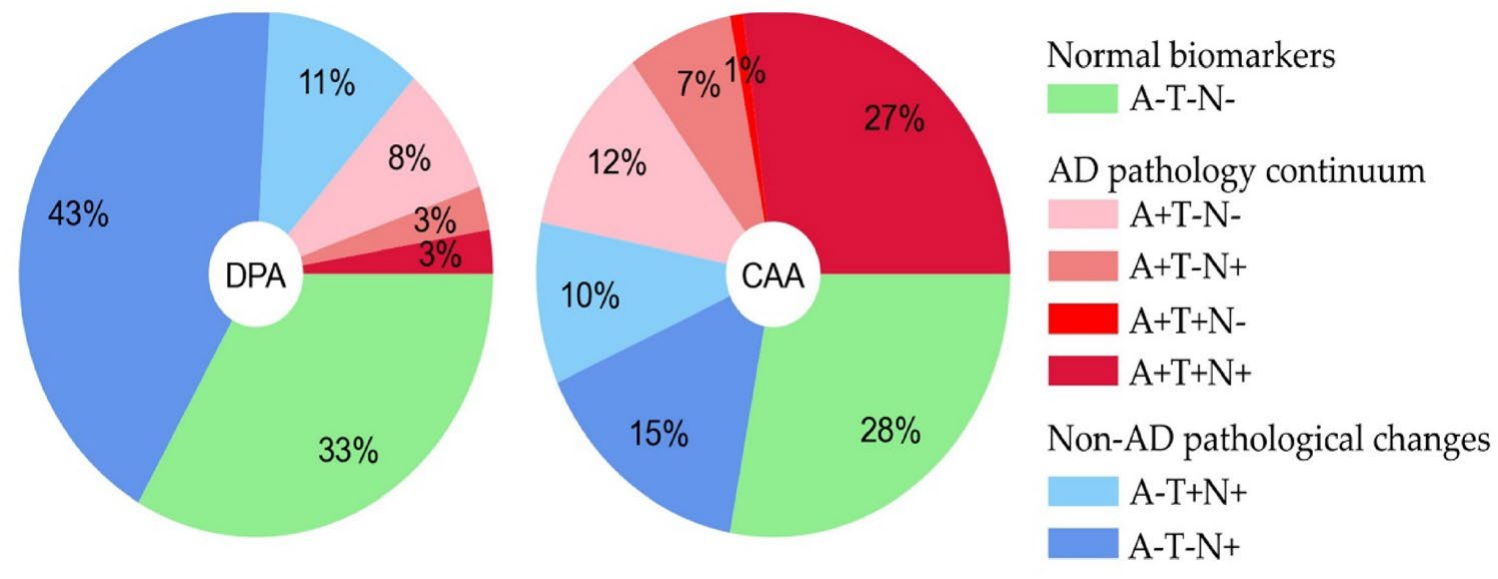
Relative frequencies of all ATN profiles in patients with DPA and CAA. None of the patients were A-T + N-. *A* amyloid-beta, *AD* Alzheimer’s disease, *CAA* cerebral amyloid angiopathy, *DPA* deep perforator arteriopathy, *T* phosphorylated tau, *N* neurodegeneration

According to the ATN classification, 56 (30%) CSVD patients had normal biomarkers (A–T–N–), and 62 (33%) CSVD patients were within the AD pathology continuum (A + T ± N ±), which was less common in DPA than CAA (13% vs. 47%, p < 0.001, Table 2). Comparisons of all ATN profile constellations are shown in Fig. 1 and Supplementary Table 1.

**Table 2:** Comparison of ATN profile prevalence between DPA and CAA

	DPA n = 75	CAA n = 111	Statistical analysis
Normal biomarkers (A-T-N-)	25 (33%)	31 (28%)	χ2 = 0.62, p = .431
AD pathology continuum (A + T ± N ±)	10 (13%)	52 (47%)	χ2 = 22.62, **p < .001**
Non-AD pathological change (A-T ± N +)	40 (53%)	28 (25%)	χ2 = 15.25, **p < .001**

Data are represented as proportions. Significant p-values are marked in bold

*AD* Alzheimer's disease, *CAA* cerebral amyloid angiopathy, *DPA* Deep perforator arteriopathy

### Neurodegeneration is frequent and commonly related to non-AD pathological change in CSVD

In CSVD patients 109 (59%) were N+, of whom 67 (62%) were attributable to non-AD pathological changes, including 18 (17%) with A–T + N + and 49 (45%) with A–T–N + . The prevalence of non-AD pathological changes was higher in DPA than CAA (53% vs. 25%, p < 0.001).

### CSVD pathology on MRI and clinical phenotype are associated with pathological ATN profiles

Next, we investigated the relationship between pathological ATN profiles, CSVD pathology on MRI and clinical phenotypes in CSVD. Therefore, univariate comparisons were conducted between patients with normal biomarkers, those within the AD pathology continuum (A-T-N–-vs. A+T±N±), and those with non-AD pathological changes (A-T-N- vs. A-T±N+), respectively (Supplementary Table 2). Significant variables (p < 0.05) were entered into multivariate logistic regression analyses to explore independent predictors for each pathological ATN profile, adjusted for age and sex.

AD pathology continuum was independently associated with lobar ICH (odds ratio (OR) 7.71 [95% confidence interval 2.09, 28.36], p = 0.002), high numbers of lobar CMB (OR 6.52 [2.42, 17.58], p < 0.001), low numbers of deep CMB (OR 0.81 [0.67, 0.99], p = 0.036), increasing age (OR 1.12 [1.05, 1.20], p < 0.001) and cognitive impairment at presentation (OR 3.28 [1.23, 8.17], p = 0.017), compared to patients with normal biomarkers. The associations remained significant analyzing CAA patients only (lobar ICH: OR 14.49 [2.50, 83.88], p = 0.003; log (lobar CMB): OR 8.21 [2.24, 30.12], p = 0.001); age: OR 1.10 [1.00, 1.20], p = 0.045; cognitive impairment: OR 3.67 [1.11, 12.20], p = 0.034), whereas only age remained predictive in DPA patients (OR: 1.15 [1.02, 1.30], p = 0.024).

Non-AD pathological change was independently related to a higher MRI CSVD sum score (OR 1.53 [1.06, 2.20], p = 0.022), history of stroke (OR 3.92 [1.67, 9.22], p = 0.002) and similarly cognitive impairment at presentation (OR 4.02 [1.69, 9.53], p = 0.002), compared to patients with normal biomarkers (Table 3). In DPA patients, only the associations for history of stroke (OR 6.08 [1.77, 20.87], p = 0.004) and cognitive impairment (OR 4.26 [1.16, 15.68], p = 0.029) remained significant. In CAA patients, only the association for cognitive impairment remained significant (OR 4.73 [1.53, 15.58], p = 0.007).

**Table 3:** Multivariable logistic regression analyses of associations with pathological ATN profiles in CSVD patients

Dependent variable	Independent variable	OR (95% CI)	P-value	Model
AD pathology continuum	Age	1.12 (1.05, 1.20)	** < .001**	p < .001 F^2^ = 1.14
	Female sex	2.43 (0.90, 6.59)	.081	
	Presence of lobar ICH	7.71 (2.09, 28.36)	**.002**	
	Log (number of lobar CMB)	6.52 (2.42, 17.58)	** < .001**	
	Number of deep CMB	0.81 (0.67, 0.99)	**.036**	
	Cognitive impairment	3.28 (1.23, 8.71)	.**017**	
Non-AD pathological change	Age	0.99 (0.95, 1.03)	.573	p < .001 F^2^ = 0.36
	Female sex	0.90 (0.40, 2.05)	.808	
	MRI CSVD sum score	1.53 (1.06, 2.20)	**.022**	
	History of stroke	3.92 (1.67, 9.22)	**.002**	
	Cognitive impairment	4.02 (1.69, 9.53)	.**002**	

Age and sex were included as confounding variables and significant associations are marked bold

*CI* confidence interval, *CMB* cerebral microbleeds, *CSVD* cerebral small vessel disease, *ICH* intracerebral hemorrhage, *WMH* white matter hyperintensities

### Pathological ATN profiles negatively impact global cognition in CSVD

Data for neuropsychological tests of global cognition (MMSE) were available in a subset of n = 92 (49%) CSVD patients. In this subgroup pathological ATN profiles were related to global cognition using linear regression analysis. Both distinct groups of pathological ATN profiles were independently associated with lower MMSE scores (AD pathology continuum: B = -3.3 [95% CI -5.6, -1.0], p = 0.006; non-AD pathological change: B = -2.7 [95% CI -5.0, -0.4], p = 0.021) (Table 4**)**. The model was adjusted for age, sex, number of vascular risk factors, history of stroke and MRI CSVD sum score as confounding variables.

**Table 4:** Associations of pathological ATN profiles with global cognition in CSVD patients

Dependent variable	Independent variables	B (95% CI)	β⁠	P-value	Model
MMSE	AD pathology continuum	-3.3 (-5.6, -1.0)	-0.36	**.006**	**p < .001** R^2^_adj_ = 0.28
	Non-AD pathological change	-2.7 (-5.0, -0.4)	-0.30	**.021**	

Multivariate linear regression analysis in n = 92 (49%) CSVD patients with available data. The model included age, sex, number of vascular risk factors, history of stroke and CSVD MRI score as confounding variables

*AD* Alzheimer’s disease, *CI* confidence interval, *CSVD* cerebral small vessel disease, *MMSE* Mini Mental State Examination

## Discussion

AD pathology and neurodegeneration CSF biomarkers were investigated in symptomatic CSVD patients according to the ATN classification. Only one third of patients showed normal biomarkers (A–T–N–). Another third of patients was within the AD pathology continuum (A+T±N±), which was more frequent in CAA than DPA and associated with older age, advanced lobar hemorrhages and cognitive impairment. The final third of patients showed non-AD pathological changes (A-T±N+), which was more frequent in DPA than CAA and associated with global CSVD severity on MRI, history of stroke and similarly cognitive impairment. Both distinct groups of pathological ATN profiles were independently associated with lower global cognition, assessed via MMSE. Using CSF biomarkers, we confirm in vivo that CSVD and neurodegenerative pathologies frequently co-occur, characterizing different pathological subtypes of symptomatic CSVD with relevance for cognitive health.

### The AD pathology continuum in CSVD

Based on the CSF Aβ_42/40_ ratio we report amyloid positivity rates of 13% in DPA and 47% in CAA, which were within ranges of recent studies that reported positivity rates from 9% to 32% in DPA [13, 15, 19, 20] and from 44% to 75% in CAA [13, 14, 16, 20, 21]. The variance of prevalence rates between studies might be influenced by methodological disparities as well as patient selection criteria. First, most studies used Aβ PET imaging compared to CSF biomarkers in our study, and there are even differences between PET tracers or CSF analysis techniques. Moreover, we applied the updated Boston criteria 2.0 for patient selection, which display superior accuracy for the detection of early CAA pathology compared to version 1.5, but presumably also includes cases with less advanced pathology [22]. Comparing cases, fulfilling the Boston criteria 1.5 vs. those fulfilling Boston criteria 2.0 only, we indeed confirm lower A + rates (50% vs. 23%). There is scarce knowledge about the stages within the AD pathology continuum, i.e. A+T- vs. A+T+, in DPA or CAA. We show that the majority of A+ DPA patients displayed isolated Aβ pathology without pTau positivity, while the majority of A + CAA patients were already in advanced stages with fully developed AD pathology (A+T+). These findings might mirror the fact that DPA and AD pathology develop additively with aging, while CAA and AD pathology are mechanistically interlinked promoting faster progression of amyloid and tau accumulation. This is supported by the fact, that in the current study the association between age and AD pathology was mainly driven by DPA patients. Likewise, neuropathological data from early and late onset AD dementia showed that the presence of DPA co-pathologies (lacunar or small infarcts) were associated with advanced age, while CAA co-pathology developed independently from age [23]. Pathophysiologically, CAA results from impaired clearance of soluble Aβ from the brain, which accelerates parenchymal Aβ deposition and, hence, tau accumulation [24]. Vice versa AD pathology promotes clearance demands and, thus, CAA, overall explaining the relationship [22, 25].

The mechanistic role of DPA pathologies such as arteriolosclerosis in cognitive decline is still a matter of debate. A neuropathological study has recently suggested that arteriolosclerosis impairs cognitive health mainly through the development of infarcts rather than through the progression of AD pathology [25]. Here, deep hemorrhagic lesions that reflect DPA downstream pathologies were associated with a lower relative risk of AD pathology. This might reflect results from recent MRI studies in cognitively impaired CSVD patients, in which lacunes and severe WMH were more associated with the absence of Aβ co-pathology [21, 26]. Contrary, a large study in presymptomatic participants has shown that vascular risk factors and CSVD on MRI are indeed associated with CSF markers of advanced Aβ pathology [27]. One could speculate that this interaction is particularly important in the silent phase, when brain pathologies develop, whereas it weakens in the end-stage of disease.

CAA explained the association between lobar hemorrhages and AD pathology. In CAA, lobar hemorrhages reflect advanced disease stages, where chronic vascular Aβ exposure induces smooth muscle cell loss, creating a rupture-prone vascular segment [22]. A study in CAA patients has similarly associated lobar CMB with the AD pathology continuum [21], and further a more precise anatomical location of lobar CMB in strictly juxta- and intracortical areas was associated with Aβ accumulation in spontaneous ICH [28]. In DPA, lobar hemorrhages reflect advanced hypertensive CSVD pathology as well (but not CAA) [29], which may explain the absent association with Aβ co-pathology.

### Non-AD pathological changes

Suspected non-AD pathological changes refers to individuals with normal Aβ, but abnormal neurodegeneration biomarkers (A-T-N+ , A-T+N+) [30]. These profiles have not yet been studied in CSVD. Notably, N+ was found in 59% of CSVD patients, mainly attributable to non-AD pathological changes. N+ was based on abnormal CSF tTau and NfL, both released into the extracellular space upon neuronal injury or death. Recent studies have related NfL, WMH severity and cognitive decline in CSVD [31–37]. This knowledge as well as the absent correlation of CSF NfL and AD pathology markers in our CSVD cohort (data not shown) suggests NfL as a promising diagnostic biofluid marker that distinguishes between AD and CSVD pathology.

The A-T+N+ biomarker profile is indicative of neurodegenerative fibrillary tau tangle pathology without Aβ accumulation and might indicate primary age-related tauopathy (PART). PART is common among the elderly and can lead to cognitive impairment. The strongest neuropathological predictor for cognitive impairment in PART is CSVD co-pathology [38, 39]. While the effects of vascular Aβ are better understood, it remains uncertain how pathological tau interacts with vascular pathology. Upcoming evidence suggests that in absence of Aβ, tau induces morphological changes in blood vessels, which impairs blood flow and accelerates neurodegeneration [40]. However, further research is needed to understand how vascular pathology interacts together with tau accumulation to accelerates cognitive decline.

### Cognition

While different pathological ATN profiles appear to characterize different CSVD subtypes, they similarly relate to poor global cognition. Additive Aβ co-pathology has been associated with accelerated longitudinal cognitive decline in lacunar stroke, CAA and CSVD with preexisting cognitive impairment [16, 19, 41]. One may speculate that this particular CSVD cohort could also benefit from Aβ modifying immunotherapy. Considerations and according studies have to be taken against the background that therapeutic mechanisms rely on vascular clearance, which might be impaired in CSVD, and related side effects, i.e., amyloid-related imaging abnormalities (ARIA), have to be monitored even more carefully. Emerging data from anti-amyloid clinical trials, such as lecanemab and donanemab, further emphasize the relevance of this issue [42–44]. While these therapies have demonstrated cognitive benefits in AD, ARIA risk remains a significant concern, particularly in patients with underlying CAA. In trials, ARIA rates were notably higher in APOE4 carriers and in those with pre-existing CMB, suggesting that vascular dysfunction plays a role in treatment safety. However, the extent and incidence of ARIA vary across anti-Aβ antibody therapies likely attributing to the differing mechanisms, therapeutic properties and selectivity to amyloid confirmations [45]. Given the high prevalence of amyloid pathology in CSVD, further research is needed to assess whether certain amyloid-lowering therapies could be beneficial for cognition in selected CSVD patients, while ensuring careful monitoring of cerebrovascular complications.

So far, there are no studies on the cognitive outcome of non-AD pathological ATN profiles in CSVD, although NfL alone has been associated with cognitive decline (as explained above). Similar to AD dementia, our study confirms that neurodegeneration leads to cognitive decline regardless of etiology. Because of that, management of vascular risk factors is a key strategy to mitigate CSVD progression and its downstream impact on neurodegeneration and cognitive decline. Longitudinal studies have shown that effective antihypertensive treatment reduces WMH progression and incident cognitive impairment, suggesting a disease-modifying potential in CSVD [46–48]. Also statins, widely used for cholesterol lowering, have been suggested to exert cerebrovascular benefits with particular focus on CSVD progression and reduced cognitive decline (reviewed in [49]). Future studies should also investigate whether these pharmacological interventions are able to improve clearance mechanisms in the context mixed vascular and amyloid pathologies.

### Strengths and limitations

The main strength of our study is the use of large and clinically diverse CSVD cohort along the whole spectrum of different disease stages, which had complete information on ATN profiles and STRIVE. The approach is of translational value, as the ATN classification is easy to implement and provides bed-side information, along with the separate consideration of DPA and CAA, which have different disease trajectories and therapies. Further, the CSF Aβ_42/40_ ratio, used here, displays superior accuracy compared to CSF Aβ_42_ alone in reference to neuropathological Aβ burden and does not correlate with neuropathological CAA severity, making it an attractive biomarker to study (parenchymal) Aβ co-pathology in vivo [50].

However, our study faces limitations. The cohort is monocentric, and findings may be affected by regional differences in patient selection and clinical presentation. Additionally, there were missing data for vascular risk factors, CSF NfL and MMSE, which is based on the clinical nature of the cohort. Cognitive function was primarily assessed using the MMSE, which does not comprehensively evaluate all cognitive domains. Furthermore, MMSE data were only available for approximately half of the cohort, likely due to incomplete documentation. This limitation should be considered when interpreting the findings related to cognitive performance. Future studies with more detailed neuropsychological assessments will be necessary to better characterize cognitive impairment in CSVD and its relationship with CSF co-pathologies. We also cannot rule out that additional age-related neurodegenerative co-pathologies (e.g. alphasynucleinopathies) have contributed to non-AD pathological changes [51]. Moreover, differences in the prevalence of ATN subtypes may arise from locally established biomarker cut-offs, and our results should therefore be validated in a multicenter cohort with larger sample sizes to enhance generalizability.

## Conclusions

The development of multiple interacting brain pathologies during aging and their mutual relevance for brain health requires multiple biomarkers to better understand the complexity of brain pathologies in vivo. Further work is necessary to fully understand the interplay of vascular and neurodegenerative diseases and capture the value of co-pathology testing. Particularly with the emergence of disease-modifying therapies for neurodegenerative and cerebrovascular diseases it will be necessary to integrate interacting biomarkers for the selection of patients with greatest benefit.

## Supplementary Information

Below is the link to the electronic supplementary material.

Supplementary file1 (DOCX 54 KB)

## Data Availability

The datasets analyzed during the current study are available from the corresponding author on reasonable request.

## Abbreviations

Aβ: Amyloid-beta
AD: Alzheimer’s disease
ARIA: Amyloid-related imaging abnormalities
CAA: Cerebral amyloid angiopathy
CI: Confidence interval
CMB: Cerebral microbleeds
CON: Controls
CSF: Cerebrospinal fluid
CSVD: Cerebral small vessel disease
DPA: Deep perforator arteriopathy
ICH: Intracerebral hemorrhage
LP: Lumbar puncture
MMSE: Mini mental state examination
MRI: Magnetic resonance imaging
NfL: Neurofilament light
PART: Primary age-related tauopathy
PET: Positron electron tomography
pTau: Phosphorylated-tau
STRIVE: Standards for reporting vascular changes on neuroimaging
tTau: Total-tau
WMH: White matter hyperintensities
