# Supplementary material for: Risk factors and clinical significance of neurodegenerative co-pathologies in symptomatic cerebral small vessel disease
Source: J Neurol. 2025 Apr 18;272(5):349. doi: 10.1007/s00415-025-13087-z (PMC12008077; doi:10.1007/s00415-025-13087-z)
Supplement: Supplementary file 1 — Supplementary file1 (DOCX 54 KB) [file 415_2025_13087_MOESM1_ESM.docx]

**Supplementary Material**

**Methods**

*Clinical data and neuropsychological assessment*

Vascular risk factors were defined by prior diagnosis of arterial hypertension, dyslipidemia and type 2 diabetes or antihypertensive, lipid lowering or antidiabetic medication. Additionally, we considered clinical laboratory blood tests for dyslipidemia (total cholesterol > 5.2 mmol/L, low density lipoprotein cholesterol > 2.6 mmol/L, high density lipoprotein cholesterol < 1.0 mmol/L or triglycerides > 1.7 mmol/L) and type 2 diabetes (HbA1c ≥ 6.5% or fasting plasma glucose level ≥ 7.0 mmol/L). The median time interval between the different diagnostic measures was as follows: MRI and lumbar puncture (LP) 6 days (IQR 2 - 12); MRI and MMSE 70 days (IQR 9 - 239); LP and MMSE 85 days (IQR 7-145).

*Cerebrospinal fluid*

CSF samples were centrifuged at 4 °C, aliquoted and stored at -80 °C until analysis. Biomarker levels were determined with ELISA Kits (until 12/2019: Innotest Aβ_40_, Innotest Aβ_42_, Innotest pTau, Innotest hTauAg, Innogenetics, Ghent, Belgium; NfL Umandiagnostics, Sweden) or automated immunoassays (LUMIPULSE® G600 II, Fujirebio lnc., Japan, from 01/2020). Locally established thresholds were as follows: 0.50 for Aβ_42/40_ ratio, 70 pg/mL for pTau, 350 pg/mL for tTau, 3643 pg/mL for NfL using ELISA kits and 0.69 for Aβ_42/40_ ratio, 56 pg/mL for pTau, 404 pg/mL for tTau using immunoassays.[1, 2] In line with ATN, each participant was classified as normal (−) or abnormal (+) for “A” based on the Aβ_42/40_ ratio ([Aβ_42_ / Aβ_40_] x 10), for “T” based on pTau and for “N” based on tTau or NfL.

*MRI aquisition and analysis*

MRI was performed using a 3T (Siemens Heathineers, Erlangen, Germany; n = 116, 42%; n = 13 controls, n = 39 DPA, n = 51 CAA, n = 13 AD) or 1.5T MRI (Siemens Heathineers, Erlangen, Germany; n = 163, 58%; n = 8 controls, n = 36 DPA, n = 60 CAA, n = 59 AD). The following sequences were used to quantify CSVD markers according to Standards for Reporting Vascular Changes on Neuroimaging (STRIVE) consensus criteria [3]: T2*-weighted gradient-recalled echo for CMB and intracerebral hemorrhage (ICH) and T2-weighted fluid-attenuated inversion recovery for white matter hyperintensities (WMH) and lacunes. MRI analysis of all participants was performed by one trained investigator (MP), blinded to demographic and clinical information. The images were evaluated using Mango software for dicom images (https://ric.uthscsa.edu/mango/). Presence and number of CMB, ICH or lacunes were assessed by according to the Microbleed Anatomomic Rating Scale and the Cerebral Haemorrhage Anatomical RaTing instrument [4, 5]. Due to the substantial variability and the presence of extremely high counts of lobar CMB, we applied logarithmic transformation to the number of lobar CMB. This approach normalized the distribution and facilitated more robust statistical analysis. WMH in deep and periventricular regions were rated according to the Fazekas scale.[6] Based on a sample of 23 randomly chosen cases across all diagnostic groups the intra- and inter-rater reliability (by ACJ, a second independent and blinded rater) was excellent for all investigated variables (>0.99 for intra- and >0.79 for inter-rater reliability).

**Supplementary Tables**

**Supplementary Table 1.  Comparison of prevalence of all ATN profiles between DPA and CAA.**

| **ATN profile** | **DPA** n = 75 | **CAA** n = 111 | **Univariate** **analysis** |
| --- | --- | --- | --- |
| A-T-N- | 25 (33%) | 31 (28%) | χ2 = 0.62, p = .431 |
| A+T-N- | 6 (8%) | 13 (12%) | χ2 = 0.67, p = .412 |
| A+T-N+ | 2 (3%) | 8 (7%) | Fisher, p = .320 |
| A+T+N- | 0 (0%) | 1 (1%) | Fisher, p = 1.00 |
| A+T+N+ | 2 (3%) | 30 (27%) | Fisher, **p < .001** |
| A-T+N+ | 8 (11%) | 11 (10%) | χ2 = 0.03, p = .867 |
| A-T-N+ | 32 (43%) | 17 (15%) | χ2 = 17.26, **p < .001** |

Data are represented as proportions. Significant p-values are marked in bold.

Abbreviations: AD, Alzheimer's disease; CAA, cerebral amyloid angiopathy; DPA, Deep perforator arteriopathy.

**Supplementary Table 2. Comparison of patient characteristics between normal and pathological biomarker status in CSVD.**

|  | **Normal biomarkers (A-T-N-)** N = 56 | **AD pathology continuum (A+T±N±)** N = 62 | **Non-AD pathological change (A-T±N+)**  N = 68 |
| --- | --- | --- | --- |
| **Demographics** |  |  |  |
| Age | 72 (65-78) | 79 (75-83) ** | 72 (64-78) |
| Female sex | 21 (38%) | 31 (50%) | 24 (36%) |
| Years of education | 13 (11-16) | 13 (11-17) | 13 (11-16) |
| **Vascular risk factors** |  |  |  |
| Hypertension | 48/51 (94%) | 49/58 (85%) | 61/66 (93%) |
| Diabetes | 17/51 (33%) | 14/58 (24%) | 20/66 (30%) |
| Dyslipidemia | 20/51 (39%) | 26/58 (45%) | 30/66 (46%) |
| **Clinical characteristics** |  |  |  |
| Cognitive impairment | 19 (34%) | 42 (68%) ** | 40 (59%) ** |
| History of stroke | 17 (30%) | 27 (44%) | 39 (57%) ** |
| History of seizure | 12 (21%) | 13 (21%) | 17 (25%) |
| Gait disturbances | 17 (30%) | 15 (24%) | 23 (34%) |
| **CSVD pathology on MRI** |  |  |  |
| CSVD MRI score | 3 (2-4) | 4 (3-4) | 4 (3-5) ** |
| CAA etiology | 31 (55%) | 52 (84%) ** | 28 (41%) |
| DPA etiology | 25 (45%) | 10 (16%) ** | 40 (59%) |
| Presence of lobar ICH | 7 (13%) | 19 (31%) * | 12 (18%). |
| Presence of deep ICH | 1 (2%) | 0 (0%) | 3 (4%) |
| Presence of cSS | 9 (16%) | 15 (24%) | 9 (13%) |
| Number of lobar CMB | 4 (2-8) | 8 (3-45) ** | 6 (1-19) |
| Log (number of lobar CMB) | 0.70 (0.48-0.95) | 0.93 (0.60-1.66) ** | 0.85 (0.35-1.30) |
| Number of deep CMB | 0 (0-1) | 0 (0-0) ** | 1 (0-4) * |
| Periventricular WMH | 3 (2-3) | 3 (2-3) | 3 (2-3) |
| Deep WMH | 2 (1-3) | 3 (2-3) | 3 (2-3) ** |
| Presence of lobar lacune | 19 (34%) | 15 (25%) | 32 (47%) |
| Presence of deep lacune | 19 (34%) | 10 (16%) * | 25 (37%) |

* p-value < .05 or ** p-value < .01 compared to individuals with normal biomarkers

Abbreviations: CAA, cerebral amyloid angiopathy; CMB, cerebral microbleeds; CSVD, cerebral small vessel disease; DPA, deep perforator arteriopathy; ICH, intracerebral hemorrhage; WMH, white matter hyperintensities

References

1. Perosa V, Priester A, Ziegler G et al. (2020) Hippocampal vascular reserve associated with cognitive performance and hippocampal volume. Brain 143:622–634. https://doi.org/10.1093/brain/awz383

2. Körtvelyessy P, Heinze HJ, Prudlo J et al. (2018) CSF Biomarkers of Neurodegeneration in Progressive Non-fluent Aphasia and Other Forms of Frontotemporal Dementia: Clues for Pathomechanisms? Front Neurol 9:504. https://doi.org/10.3389/fneur.2018.00504

3. Wardlaw JM, Smith EE, Biessels GJ et al. (2013) Neuroimaging standards for research into small vessel disease and its contribution to ageing and neurodegeneration. Lancet Neurol 12:822–838. https://doi.org/10.1016/S1474-4422(13)70124-8

4. Gregoire SM, Chaudhary UJ, Brown MM et al. (2009) The Microbleed Anatomical Rating Scale (MARS): reliability of a tool to map brain microbleeds. Neurology 73:1759–1766. https://doi.org/10.1212/WNL.0b013e3181c34a7d

5. Charidimou A, Schmitt A, Wilson D et al. (2017) The Cerebral Haemorrhage Anatomical RaTing inStrument (CHARTS): Development and assessment of reliability. J Neurol Sci 372:178–183. https://doi.org/10.1016/j.jns.2016.11.021

6. Wahlund LO, Barkhof F, Fazekas F et al. (2001) A new rating scale for age-related white matter changes applicable to MRI and CT. Stroke 32:1318–1322. https://doi.org/10.1161/01.str.32.6.1318
